# A Novel Deep-Learning-Based Framework for the Classification of Cardiac Arrhythmia

**DOI:** 10.3390/jimaging8030070

**Published:** 2022-03-10

**Authors:** Sonain Jamil, MuhibUr Rahman

**Affiliations:** 1 Department of Electronics Engineering, Sejong University, Seoul 05006, Korea; sonainjamil@sju.ac.kr; 2 Department of Electrical Engineering, Polytechnique Montreal, Montreal, QC H3T 1J4, Canada

**Keywords:** cardiac arrhythmia, ECG, deep learning, attention block, heart disease, features extraction

## Abstract

Cardiovascular diseases (CVDs) are the primary cause of death. Every year, many people die due to heart attacks. The electrocardiogram (ECG) signal plays a vital role in diagnosing CVDs. ECG signals provide us with information about the heartbeat. ECGs can detect cardiac arrhythmia. In this article, a novel deep-learning-based approach is proposed to classify ECG signals as normal and into sixteen arrhythmia classes. The ECG signal is preprocessed and converted into a 2D signal using continuous wavelet transform (CWT). The time–frequency domain representation of the CWT is given to the deep convolutional neural network (D-CNN) with an attention block to extract the spatial features vector (SFV). The attention block is proposed to capture global features. For dimensionality reduction in SFV, a novel clump of features (CoF) framework is proposed. The k-fold cross-validation is applied to obtain the reduced feature vector (RFV), and the RFV is given to the classifier to classify the arrhythmia class. The proposed framework achieves 99.84% accuracy with 100% sensitivity and 99.6% specificity. The proposed algorithm outperforms the state-of-the-art accuracy, F1-score, and sensitivity techniques.

## 1. Introduction

**Figure 1:**
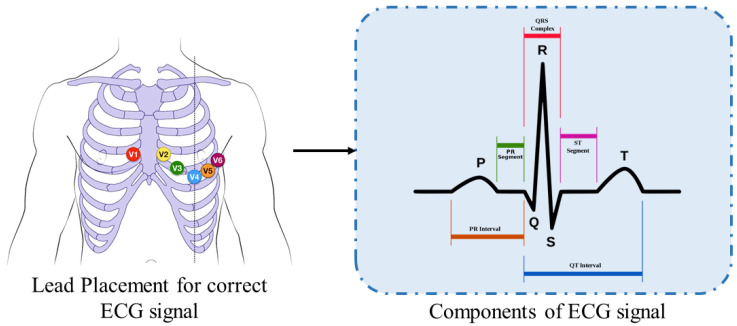
ECG lead placement with components of ECG beat.

Cardiovascular diseases (CVDs) are the primary cause of death every year. According to the World Health Organization (WHO), approximately 17.9 million people died due to CVD in 2019, which translated as CVDs causing 32% of global deaths. Of these deaths, 85% were due to heart attacks [1]. A heart attack is caused by the blockage of one or more coronary arteries, whereas other CVDs cause the remaining 15% of the deaths due to cardiac arrhythmia. Cardiac arrhythmia is an irregular heartbeat. ECGs are used to monitor heartbeats. The ECG signal is recorded through electrocardiography in which 12 leads are placed on the body. For correct recording of the ECG, the placement of the leads is very important [2]. The correct positions of the leads and the components of the ECG signal are shown in Figure 1.

A normal heartbeat has a regular rhythm in the ECG plot, whereas arrhythmia heartbeats have irregularities. In this article, sixteen classes of cardiac arrhythmia are considered. These classes are atrial premature beats (APBs), atrial flutter (AFL), atrial fibrillation (AFIB), supraventricular tachyarrhythmias (SVTA), Wolff–Parkinson–White (WPW), premature ventricular contraction (PVC), idioventricular rhythm (IVR), ventricular bigeminy, ventricular trigeminy, left bundle branch block (LBBBB), ventricular tachycardia (VT), ventricular flutter (VFL), second-degree heart block (SDHB), the fusion of ventricular and regular beat, right bundle branch block (RBBBB) and short PR [3]. Not all cardiac arrhythmias are harmful, but a few are, such as AFIB and VFL, which can cause heart attacks. Thus, it is necessary to identify the arrhythmia class to avoid potential damage.

The classification of ECG beats into arrhythmic cardiac classes has been performed using several conventional and deep-learning-based algorithms. ECG is a one-dimensional (1D) signal; therefore, the anomalies can be detected using machine learning approaches. Prasad et al. [4] used k-nearest neighbors (kNNs) to classify arrhythmia and achieved a 97.65% accuracy. The framework only considered the nonlinear local features and 1D signals. Similarly, the authors of [5] used kNNs to detect arrhythmia and achieved a sensitivity of 97.22% for the detection. The method only considered the handcrafted features of the 1D signals. With the advancements in deep learning, many deep learning architectures have been proposed to classify arrhythmias. In [6], the authors proposed a multilayer perceptron (MLP) and convolutional neural network (CNN)-based architecture for the classification of arrhythmia using 1D ECG signals. They achieved an accuracy of 88.7% for the MLP and 83.5% for CNN-based framework. Similarly, ref. [7] converted an ECG signal to a 2D signal and used a CNN-based approach to classify arrhythmia. They only considered eight classes and achieved a classification accuracy of 99.11%.

Inspired by this, we propose a novel deep-learning-based framework for classifying cardiac arrhythmia using attention-block-based CNN and the clump of features (CoF). The ECG signal is segmented into beats and converted into a 2D time–frequency domain signal using CWT. The features are extracted using attention-based CNN. After feature extraction, feature reduction is achieved with the help of the CoF model; then, classification is performed using classifiers. The performance of several classifiers is compared.

The rest of the article is organized as follows: Section 2 presents related work; the proposed methodology is explained in Section 3; Section 4 shows the experimental results; and in Section 5, conclusions are drawn.

## 2. Related Work

ECG signals are widely used for the detection and diagnosis of heart diseases. The signals can also be used for the classification of arrhythmia. These signals are one-dimensional and can be transformed into two-dimensional signals to classify the arrhythmia beats. ECG-based arrhythmia classification can be performed using handcrafted and deep temporal features. In [8], the authors proposed a gray-level co-occurrence matrix (GLCM) and gray-level run-length matrix (GLRLM)-based model for the classification of arrhythmia. They used six machine learning classifiers and achieved 90.42% accuracy for the 1D signal. In this framework, only handcrafted features are considered.

Similarly, in [9], a multi-resolution representation-based deep neural network model is proposed to classify arrhythmia. This model achieved an F1-score of 0.9238. The model is only applicable for the 1D signal and only considers handcrafted features.

In [10], an attention-based model is proposed for the classification of arrhythmia. The authors used the PhysioNet public dataset and achieved 92.8% accuracy, but the ECG signal in 1D is considered classification. In [11], the authors calculated RR intervals to detect arrhythmia. The approach uses conventional methods and achieves an accuracy of 99.98%. They also considered ECG in 1D.

In [12], the authors converted the ECG signal into a 2D spectrogram and proposed a framework based on the spectrogram for arrhythmia classification. They achieved an accuracy of 99.02% for the 2D-based CNN model. They only considered seven classes of arrhythmia. In [13], the authors demonstrated a support vector machine (SVM)-based framework for classifying arrhythmia using a 1D ECG signal. They considered 17 classes of arrhythmia and achieved 97.3% accuracy. The authors of [14] used YOLO to detect arrhythmia, and they considered only four classes of arrhythmia. Similarly [15], proposed a CNN-based framework for classifying cardiac arrhythmia using short-time Fourier transform (STFT) as input signal and achieved 99.00% accuracy, but they only considered four arrhythmia classes.

Furthermore, SVM is used to classify arrhythmia using the MIT-BIH database in [16]. They used selective bands and achieved 97.06% accuracy. Similarly, the authors of [17] used SVM to classify arrhythmia into four classes and achieved an accuracy of 83%. Moreover, in [18], the authors demonstrated cardiac arrhythmia classification using fog computing. They used the LSTM network for the classification of cardiac arrhythmia. Majahad et al. [19] demonstrated the conversion of ECG into time–frequency domain representation to classify arrhythmia. They used bagging methods and achieved 99% accuracy.

Motivated by the conversion of ECG into the 2D signal, we propose a novel deep-learning-based framework for classifying sixteen cardiac arrhythmia classes. A plethora of pre-trained D-CNNs, such as AlexNet [20], ResNet-50 [21], VGG-19 [22], Inception v3 [23], GoogLeNet [24], ShuffleNet [25], SqueezeNet [26], EfficientNetb0 [27], Xception [28], and DarkNet-53 [29], as well as the novel attention-based CNN ArrhythmiaNet, have been used for the feature extraction of 2D time–frequency representations of ECG beats. These features are reduced, and the classifier is trained using reduced features. The upcoming section explains the proposed methodology.

## 3. Proposed Methodology

**Figure 2:**
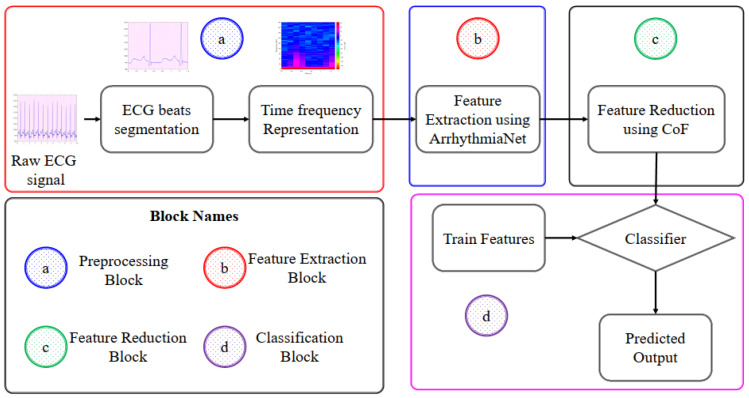
Block diagram of the proposed framework.

The proposed framework consists of four major blocks. In the first block, the ECG signal is segmented into beats and then converted into 2D time–frequency representation using CWT. These 2D signals are given to the second block where the local, as well as global features, are extracted using a novel ArrhythmiaNet architecture. The output of ArrhythmiaNet is SFV. SFV is fed into the third block where the k-means clustering algorithm is applied, and the features are reduced to obtain RFV. This RFV is used to train the classifier in the fourth block, and the outputs of the classifier are the arrhythmia classes. The proposed framework is illustrated in Figure 2.

### 3.1. Preprocessing

We used the MIT-BIH dataset [30] for this research. The dataset consists of ECG signals of the standard and cardiac arrhythmia classes, and there are a total of 16 classes of cardiac arrhythmia. Table 1 shows the number of samples in each class. Each sample has 1000 fragments.

**Figure 3:**
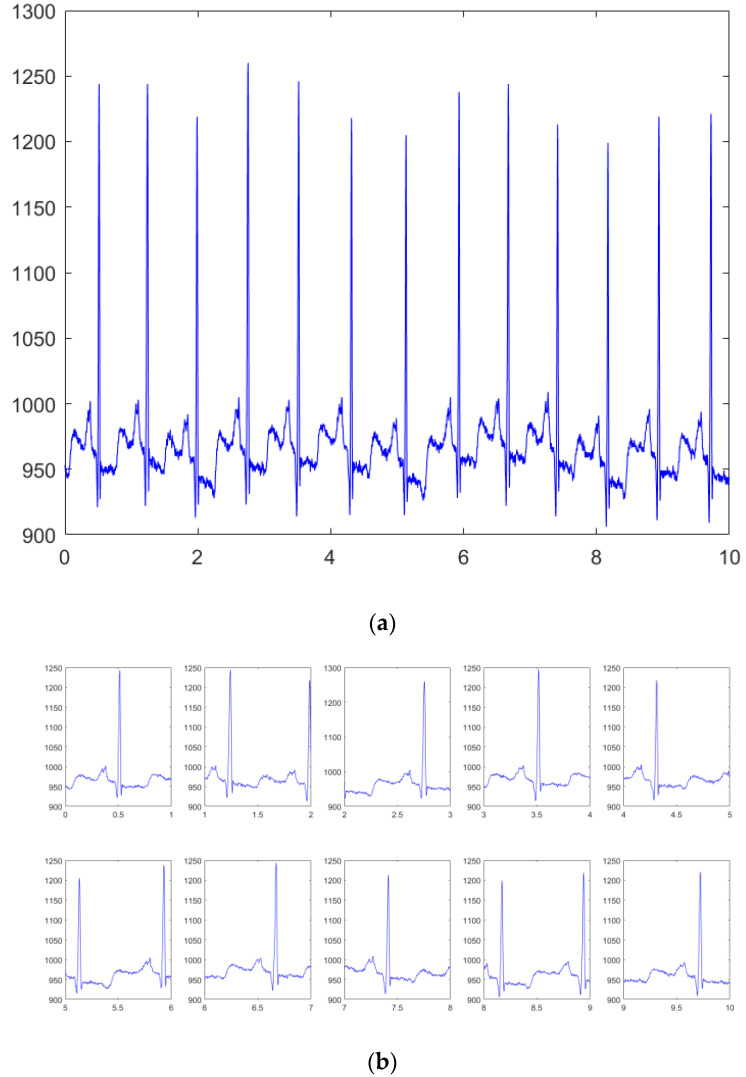
Complete ECG signal with its segmented beats: (**a**) complete ECG signal; (**b**) segmented beats.

All the ECG signals are segmented into beats using a timeframe window. The timeframe is kept constant for all ECG signals. Each ECG signal of ten seconds is divided into ten beats of one second. Figure 3 shows the ECG signal and its segmented beats.

After segmentation, the segmented beats are converted into the 2D time–frequency domain representation. For 2D time–frequency domain representation, we have used the CWT method. Traditional arrhythmia detection models extract features from the 1D ECG signal, which causes abortive classification results and low accuracy. We use CWT to transform a 1D signal into a 2D signal for better signal analysis and features extraction.

#### Continuous Wavelet Transform (CWT)

CWT expresses the signals in the form of wavelet functions. These wavelet functions are localized in the time domain as well as the frequency domain. By allowing the translation and scaling parameters of the wavelets to change constantly, CWT yields a comprehensive representation of the 1D signal. The time–frequency domain representation $ECG_{\omega }\left(\alpha ,\beta \right)$ of a continuous ECG beat $ecg_{beat}\left(t\right)$ can be expressed as:

(1) $$ECG_{\omega }\left(\alpha ,\beta \right)=\frac{1}{\sqrt{\left|\alpha \right|}}\int_{-\infty }^{\infty }ecg_{beat}\left(t\right)\bar{\Psi }\left(\frac{t-\beta }{\alpha }\right)dt,\;where\;\alpha ,\beta \;\in \mathbb{R}$$

Here, α is the scaling parameter and should be greater than 0; *β* is the translation parameter; *t* is the instant of time; $\bar{\Psi }\left(t\right)$ is the continuous mother wavelet. $\bar{\Psi }\left(t\right)$ provides the translation and scaling of the original wavelet $ecg_{beat}\left(t\right)$. The original ECG beats can be expressed as:

(2) $$ecg_{beat}\left(t\right)=C_{\Psi }^{-1}\overset{\infty }{\underset{-\infty }{\int }}\overset{\infty }{\underset{-\infty }{\int }}ECG_{\omega }\left(\alpha ,\beta \right)\frac{1}{\sqrt{\left|\alpha \right|}}\tilde{\Psi }\left(\frac{t-\beta }{\alpha }\right)ab\frac{da}{a^{2}},$$

where $C_{\Psi }$ is the wavelet constant whose value is 0 < $C_{\Psi }$ < ∞ and can be written as shown in Equation (3).

(3) $$C_{\Psi }=\overset{\infty }{\underset{-\infty }{\int }}\frac{\bar{\hat{\Psi }}\left(\omega \right)\hat{\tilde{\Psi }}\left(\omega \right)}{\left|\omega \right|}d\omega ,$$

The integration of the admissible wavelet must be zero. For this, $ecg_{beat}\left(t\right)$ is recovered using the second inverse wavelet transform, as shown in Equation (2).

(4) $$ecg_{beat}\left(t\right)=\frac{1}{2\pi \bar{\hat{\Psi }}\left(1\right)}\overset{\infty }{\underset{-\infty }{\int }}\overset{\infty }{\underset{-\infty }{\int }}\frac{1}{\alpha^{2}}ECG_{\omega }\left(\alpha ,\beta \right)e^{\left(\frac{\iota \left(t-\beta \right)}{\alpha }\right)}d\beta d\alpha ,$$

The instantaneous wavelet at time *t* is defined as:

(5) $$\Psi \left(t\right)=\omega \left(t\right)e^{\iota t}$$

where $\omega \left(t\right)$ is the window. We derive the time–frequency representation of the beats by using a filter bank in CWT. All the 2D representations are resized to 256×256 and divided into training and testing using tenfold cross-validation.

### 3.2. Train Test Dataset Split

After preprocessing, the dataset is divided into training and testing. Training involves 70% of the preprocessed data, whereas testing contains 30% of the preprocessed data.

### 3.3. Features Extraction Using ArrhythmiaNet

**Figure 4:**
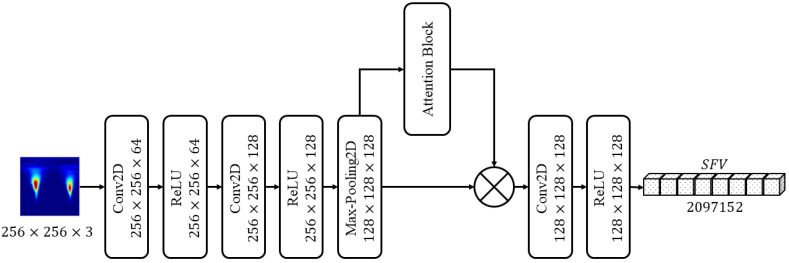
Proposed ArrhythmiaNet with the attention block.

After preprocessing, the features of the 2D representations are extracted with the help of a novel ArrhythmiaNet with an attention block. ArrhythmiaNet consists of three 2D convolutional layers with the rectified linear unit (ReLU) as the activation function, one max-pooling layer, attention block, one flatten layer, and one last fully connected layer. The output of the fully connected layer is called the spatial feature vector. The attention block is explained in Section 3.3.1. The output of this attention block is multiplied with the max-pooling layer of the main ArrhythmiaNet. The purpose of this attention block is to capture global features, as explained in [31]. The architecture of the ArrhythmiaNet with the attention block is shown in Figure 4.

The vector from the proposed CNN is called the spatial feature vector (SFV). There are 2097152 full features in the SFV. After feature extraction, these features are reduced to 4096 using a clump of features (CoF) module.

#### 3.3.1. Attention Block

In the attention block, a self-attention mechanism is followed. In particular, the self-attention block calculates the response at one position in the feature map as a weighted sum of the features from all positions. As a result, the weights are calculated with only a small computational cost.

**Figure 5:**
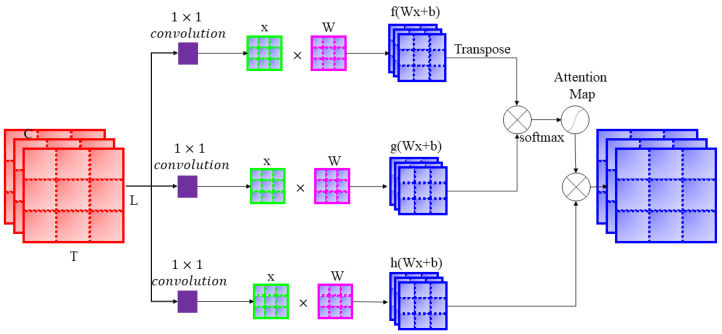
Attention block.

Figure 5 shows the attention block. In the attention block, the input features map denoted by x is transformed into three feature spaces, *f*, *g* and *h.* To derive these feature spaces, initially, the input is passed through 1×1 convolution, and then it is multiplied by the trained weighted matrices *W* using the following equations.

(6) $$f\left(x_{i}\right)=Wx_{i}+b$$

(7) $$g\left(x_{j}\right)=Wx_{j}+b$$

(8) $$h\left(x_{i}\right)=Wx_{i}+b$$

where *W* denotes the weights and *b* denotes the bias parameters. Then, matrices $f{\left(x_{i}\right)}^{T}$(transpose of $f\left(x_{i}\right)$) and matrices $g\left(x_{j}\right)$ are multiplied by the softmax function and the weighted value vectors are summed to obtain the attention map. Then, attention map and matrix $h\left(x_{i}\right)$ are multiplied to derive the attention feature matrix. The attention mechanism used is similar to the self-attention mechanism used in [32]. However, we have not used linear regulizers.

### 3.4. Feature Reduction Using CoF

The reduced feature vector (RFV) is obtained using the CoF method, as explained in [33]. In CoF, k-means clustering is applied to the SFV to derive the vocabulary of features. k-means clustering is a highly unstable algorithm; therefore, the optimal value of k to make clusters is crucial. The optimal value of the clusters can be obtained with the help of the elbow method, in which the sum of the squared errors is plotted against different values of k, and the optimal value of k is selected. From the elbow plot, we obtained the value of k as 10. We further validated the clusters using silhouette analysis (SA). The pseudo-code of SA is presented in Algorithm 1.

**Algorithm 1:** Silhouette Analysis (SA)For every sample   1.Find the mean distance from all features in the same cluster $\left({𝒶}_{i}\right)$  2.Find the mean distance from all features in the closest cluster $\left({𝒷}_{i}\right)$  3.Find the coefficient: ${𝒮}_{𝒸}=\frac{{𝒷}^{i}-{𝒶}^{i}}{max({𝒶}^{i},{𝒷}^{i})}$
**If**${𝒮}_{𝒸}=0$, the sample is very close to the neighboring clusters**If**${𝒮}_{𝒸}=1$, the sample is far away from the neighboring clusters**If**${𝒮}_{𝒸}=-1$, the sample is assigned to the wrong clusters


**Figure 6:**
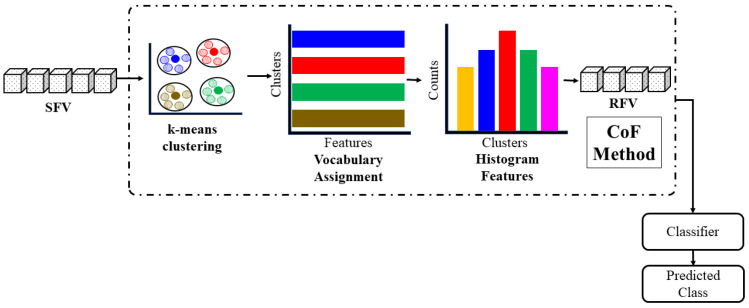
Features reduction with the clump of features (CoF) method.

After clustering, the histogram of the clustered vocabularies is drawn to derive the RFV. The RFV has the most important features. In feature reduction, the training and testing features are treated separately because the inter-patient paradigm is more strict than the intra-patient paradigm. The CoF method is illustrated in Figure 6.

After feature reduction, the RFV is given to the classifier for the classification of features into cardiac arrhythmia classes.

### 3.5. Classification

We used a support vector machine (SVM) for classification [34,35]. An SVM classifies objects by drawing a hyperplane and support vectors. The margin between the support vectors demonstrates the performance of the classifier. The greater the margin of the support vectors, the better the performance of the classifier, and vice versa. We have trained our SVM using RFV of the training dataset, and the RFV of the test dataset was used for performing the classification.

The upcoming section explains the experimental results of the proposed framework.

## 4. Experimental Results

The proposed framework is evaluated in terms of accuracy, sensitivity, specificity, F1-score, and classification error. Accuracy of the model was calculated using Equation (9).

(9) $$Accuracy=\frac{TP+TN}{TP+TN+FP+FN}$$

where TP and TN denote true positive and true negative, respectively, whereas FP and FN denote false positive and false negative, respectively. The accuracy of the system shows its ability to correctly classify arrhythmia classes. Specificity is the ratio of the prediction of normal beats present in the case of binary classification and can be calculated using Equation (10).

(10) $$Specificity\;\left(S_{P}\right)=\frac{TN}{TN+FP}$$

The ratio of the prediction of abnormal or cardiac arrhythmia class is called the sensitivity of the model and can be determined using Equation (11).

(11) $$Sensitivity\;\left(S_{E}\right)=\frac{TP}{TP+FN}$$

The F1-score is the mean of the specificity and sensitivity; the formula to calculate the F1-score is given in Equation (12).

(12) $$F1-score=\left[\frac{\left(S_{E}*S_{P}\right)}{\left(S_{E}+S_{P}\right)}\right]*2$$

Here, $S_{E}$ and $S_{P}$ denote sensitivity and specificity, respectively. We also considered Cohen’s kappa for the evaluation of our model. This can be calculated using Equation (13).

(13) $$Cohen’s\;Kappa\;\left(\kappa \right)=\left[\frac{\left(TP.TN-FP.FN\right)}{\left(TP+FP\right).\left(FP+TN\right)+\left(TP+FN\right).\left(FN+TN\right)}\right]*2$$

The optimal simulation parameters for training of the 36 million parameters of ArrhythmiaNet are shown in Table 2.

The performance of ArrhythmiaNet was compared with the pre-trained D-CNNs, and is shown in Table 3.

**Figure 7:**
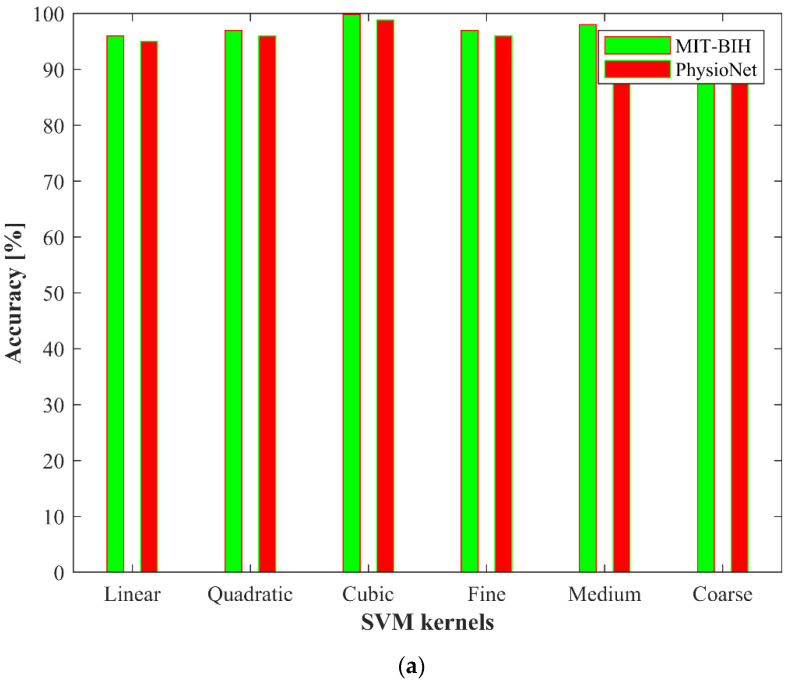

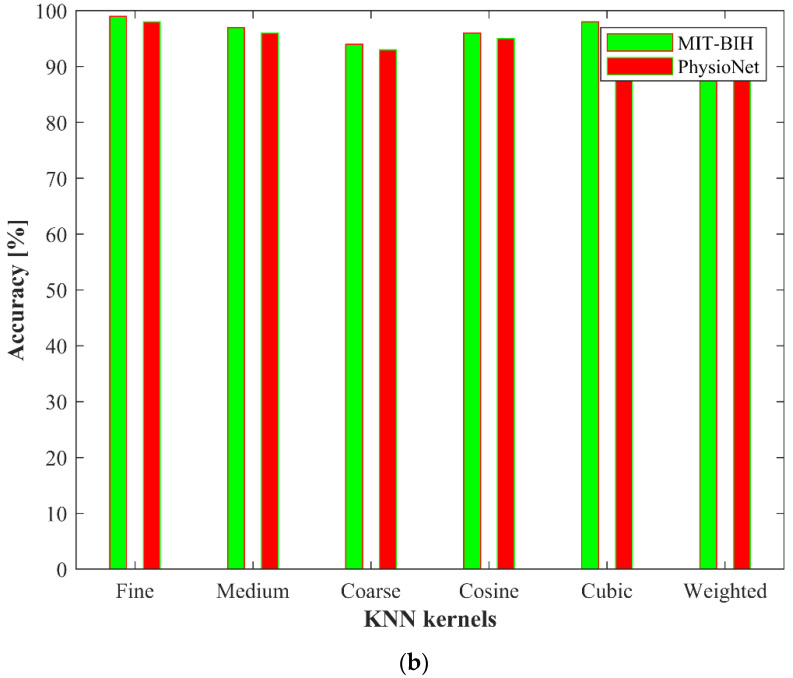
Performance of the different kernels of both classifiers on MIT-BIH and PhysioNet datasets: (**a**) SVM classifier; (**b**) kNN classifier.

From Table 3, the performance of the proposed ArrhythmiaNet based framework is better than the other pre-trained D-CNNs. We have also compared the performance of the proposed ArrhythmiaNet with the PhysioNet dataset. The performance of the different kernels of SVM and kNN for the MIT-BIH and PhysioNet datasets is shown in Figure 7.

We have also compared the accuracy of ArrhythmiaNet with the existing models. This comparison is presented in Table 4.

From Table 4, all the CNN-based models which used ECG as 2D signals considered a maximum of eight classes of cardiac arrhythmia, and the maximum accuracy achieved by [7] was 99.11%. We have considered seventeen classes of arrhythmia and achieved 99.84%, which outperforms all the existing methods.

**Figure 8:**
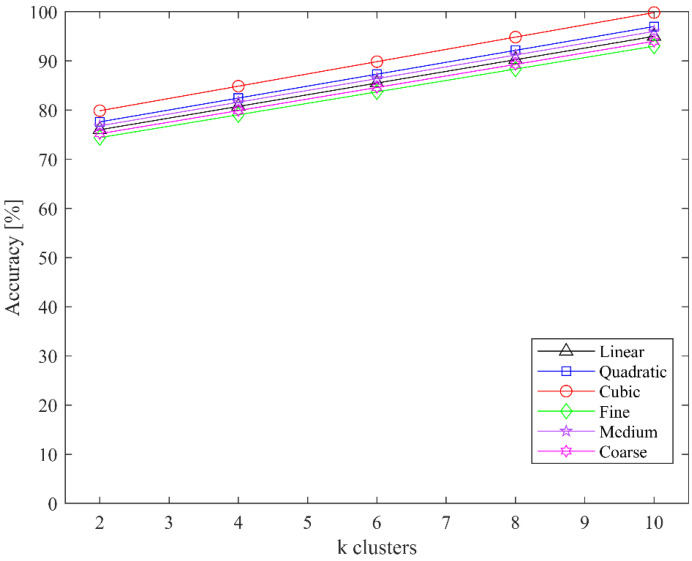
Classification accuracy of SVM for different values of k clusters.

**Figure 9:**
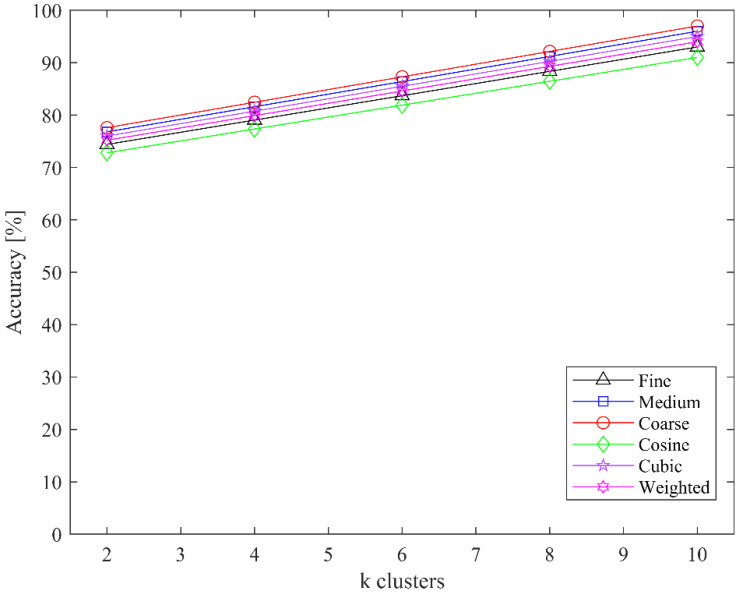
Classification accuracy of kNN for different values of k clusters.

We also evaluated the classification accuracy of both classifiers for the MIT-BIH dataset for different clusters in the CoF module; the highest accuracy was achieved at 10 clusters. The classification accuracies of SVM and kNN for different clusters are shown in Figure 8 and Figure 9, respectively.

**Figure 10:**
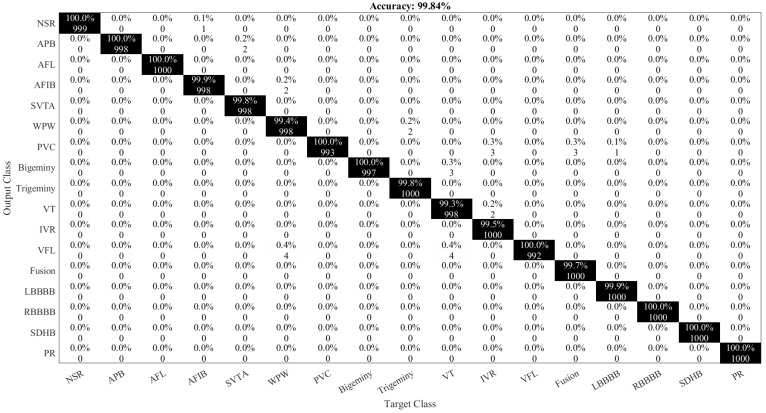
Confusion matrix of the ArrhythmiaNet with SVM classifier.

The confusion matrix of ArrhythmiaNet using the SVM classifier is shown in Figure 10.

### 4.1. Ablation Study

**Figure 11:**
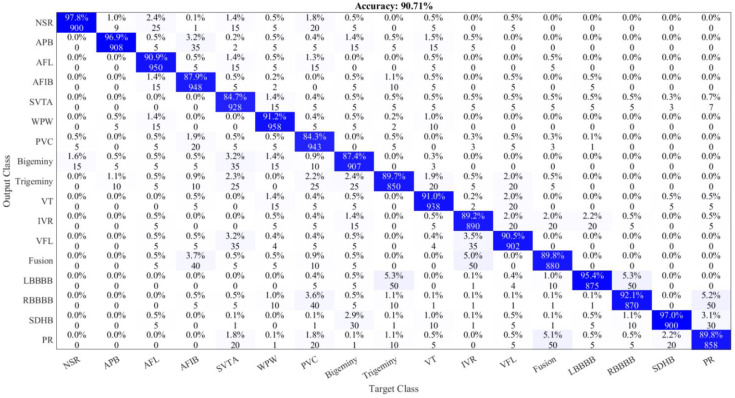
Confusion matrix of ArrhythmiaNet without the attention block.

We performed an ablation study to show the impact of the attention block added in ArrhythmiaNet. Initially, we removed the complete block and calculated the performance metrics of the network. Then, we added the block and calculate the performance metrics. The accuracy of ArrhythmiaNet without the attention block was 90.71%, which is significantly lower than the other D-CNN models. However, when attention model block was added, more detailed features were extracted, and the accuracy of the network was 99.84%, which outperformed the other D-CNNs models. Figure 11 shows the confusion matrix of ArrhythmiaNet without the attention block.

### 4.2. Limitations and Future Directions

The proposed framework is dependent on the attention block, and the accuracy of the model decreases if the attention block is removed. The performance of the model also varies by selecting different features. The inter-patient paradigm is also a factor which affect the performance of the model. These limitations can be resolved by the use of class tokens and the multi attention heads similar to the vision transformers. In future, we plan to use a hybrid model of vision transformers to categorize different classes of arrhythmic cardiac and to resolve these limitations.

Section 5 presents a brief conclusion of this research.

## 5. Conclusions

Cardiovascular diseases are the primary cause of death. Most deaths are due to heart attacks. An irregular heartbeat causes a heart attack. A normal heartbeat has rhythm in the ECG plot, whereas an abnormal heartbeat shows an irregular ECG plot. These irregular ECGs are called cardiac arrhythmia. The timely classification of these cardiac arrhythmias can avoid potential damage. In this article, we propose the novel attention-based ArrhythmiaNet with a CoF module to categorize seventeen classes of heartbeats. The proposed method achieved 99.84% accuracy with an SVM classifier. The sensitivity of the proposed technique was 100%, and the F1-score was 99%. The classification accuracy of ArrhythmiaNet with a kNN classifier was 98.64%, which is inferior to SVM. We have also compared the proposed framework with existing methods, and the experimental results verify that ArrhythmiaNet outperforms all the existing techniques in terms of accuracy.

## Figures and Tables

**Table 1:** Summary of dataset classes.

Class Name	Number of Samples
NSR	283
APB	66
AFL	20
AFIB	135
SVTA	13
WPW	21
PVC	133
Bigeminy	55
Trigeminy	13
VT	10
IVR	10
VFL	10
Fusion	11
LBBBB	103
RBBBB	62
SDHB	10
PR	45

**Table 2:** Summary of hyperparameters for ArrhythmiaNet training.

Parameter Name	Parameter Value
Optimizer	Stochastic Gradient Descent (SGD)
Momentum	0.9
Learning Rate	0.001
Mini batch size	32
Learning rate decay	10^−7^
Loss function	Cross entropy

**Table 3:** Comparison of the testing performance of the different D-CNNs with ArrhythmiaNet.

Name of D-CNN	Classifier	Accuracy	F1-Score	Sensitivity	Specificity	Cohen’s Kappa	Error
AlexNet [20]	SVM	98.70%	0.968	97.10%	96.50%	0.950	1.30%
ResNet-50 [21]	SVM	95.40%	0.951	94.60%	95.60%	0.930	4.60%
VGG-19 [22]	SVM	89.80%	0.918	93.3%	90.40%	0.900	10.20%
Inception v3 [23]	SVM	98.20%	0.951	94.6%	95.60%	0.930	1.80%
GoogLeNet [24]	SVM	94.40%	0.930	92.50%	93.50%	0.910	5.60%
ShuffleNet [25]	SVM	96.80%	0.964	95.40%	97.40%	0.940	3.20%
SqueezeNet [26]	SVM	86.83%	0.918	93.30%	90.40%	0.890	13.17%
EfficientNetb0 [27]	SVM	96.50%	0.996	100%	99.20%	0.980	3.50%
Xception [28]	SVM	98.55%	0.996	100%	99.20%	0.980	1.45%
DarkNet-53 [29]	SVM	96.35%	0.928	92.3%	93.30%	0.900	3.65%
**ArrhythmiaNet**	**SVM**	**99.84%**	**0.998**	**100%**	**99.60%**	**0.990**	**0.16%**

**Table 4:** Comparison of the performance of ArrhythmiaNet with the state-of-the-art techniques.

Ref.	Model	ECG Signal	Arrhythmia Classes	Accuracy
[4]	kNN	1D	03	97.65%
[5]	kNN	1D	17	97.22%
[6]	MLP	1D	09	88.7%
[7]	CNN	2D	08	99.11%
[8]	GLCM	1D	06	90.42%
[10]	Attention-based CNN	1D	07	92.8%
[12]	CNN	2D	08	99.02%
[13]	SVM	1D	17	97.3%
[15]	CNN	2D	04	99.00%
[16]	SVM	1D	04	97.06%
[17]	SVM	1D	04	83.00%
**This paper**	**ArrhythmiaNet**	**2D**	**17**	**99.84%**

## Data Availability

Dataset link: https://doi.org/10.17632/7dybx7wyfn.3 (accessed on 1 March 2022).
